# Freezability biomarkers in bull epididymal spermatozoa

**DOI:** 10.1038/s41598-019-49378-5

**Published:** 2019-09-05

**Authors:** Do-Yeal Ryu, Won-Hee Song, Won-Ki Pang, Sung-Jae Yoon, Md Saidur Rahman, Myung-Geol Pang

**Affiliations:** 1 Department of Animal Science and Technology, Chung-Ang University, Anseong, Gyeonggi-do, 17546 Republic of Korea; 2 BET Research Institute, Chung-Ang University, Anseong, Gyeonggi-do, 17546 Republic of Korea

**Keywords:** Biochemistry, Reproductive biology

## Abstract

Sperm cryopreservation is an important tool for storing genetic traits and assisted reproduction techniques. Several studies have developed semen cryopreservation protocols. However, the sperm proteome is different between ejaculated and epididymal spermatozoa and little is known about cryopreservation effects on epididymal spermatozoa. Therefore, our study aimed to (i) investigate the differences of sperm parameters based on the freezing tolerance of spermatozoa and (ii) identify potential markers to predict the freezability of bull epididymal spermatozoa. Our preliminary study demonstrated that spermatozoa from individual bulls differ in cryopreservation freezability. We categorized spermatozoa into high freezing-tolerant spermatozoa and low freezing-tolerant spermatozoa group based on sperm motility after freezing/thawing. We evaluated several sperm functional parameters, including sperm motility/motion kinematics, sperm speed parameters, viability, mitochondrial activity, and capacitation status. Our results demonstrated that motility, sperm speed parameters, viability, and mitochondrial membrane potential had significant differences between the two groups but motion kinematics and capacitation status did not. In addition, the concentration of three proteins - glutathione s-transferase mu 5, voltage-dependent anion-selective channel protein 2, and ATP synthase subunit beta, differed between both groups. Thus, our research highlighted differences in bull epididymal spermatozoa freezability upon cryopreservation and these proteins might be useful markers to select high freezing-tolerant epididymal spermatozoa.

## Introduction

For decades, cryopreservation was considered to be a remarkable tool to preserve a variety of cell types for long-term storage^1–3^. This technique has been applied in several clinical and research applications, including assisted reproductive techniques, genetic improvement, and bio-banking^3–5^.

Since sperm cryopreservation was first introduced by Polge^6^, there has been a rapid rise in the use of sperm cryopreservation in the livestock industry^7^. It is reported that bull semen cryopreservation can lead to rapid and large-scale genetic improvements with decreasing disease transduction. In addition, bull cryopreserved semen has already been commercially sold with highly successful rates of insemination compared to natural mating^7,8^. Despite its benefits, cryopreservation has a detrimental impact on sperm quality. During cryopreservation, the freezing/thawing step is one of the major factors that can causes several types of damage to spermatozoa, such as cold shock, ice crystal formation, and oxidative stress which can lead to a disruption of sperm physiology and eventually fertility^8–14^. However, some bull semen shows tolerance to this stress. Therefore, discovering a specific marker for estimating sperm freezability, such as sperm motility, membrane integrity, or DNA integrity, has become a central issue in the livestock industry^11,14,15^.

Many comprehensive proteomic studies have been successfully conducted to determine a potential marker for predicting sperm freezability in various animal species^12,13,16,17^. Several studies demonstrated that sperm freezability is highly variable in ejaculated spermatozoa. Recently, a study reported by Wang *et al*.^18^ had shown that high concentration of heat shock protein is closely related with high freezability in bull spermatozoa. In addition, another study proposed that specific sperm and seminal plasma proteins were associated with high and low freezability, respectively, in bull semen^16^. However, the ejaculated and epididymal spermatozoa consist of different proteomes that may be differently associated with sperm freezability^19,20^. Ejaculated spermatozoa undergo several functional and physiological modifications by several factors, especially seminal plasma proteins that are constructed by the caudal epididymis and accessory sexual glands during ejaculation^21,22^. Therefore, it is tempting to speculate that these different protein complexes might influence sperm freezability after ejaculation. While there is a large body of knowledge suggesting potential markers using spermatozoa from ejaculated semen, very little is known about the specific sperm bio-marker using spermatozoa from the caudal epididymis, which are not affected by seminal plasma.

Therefore, the aim of our work is i) to investigate the differences of sperm function according to freezability and ii) to discover the potential biomarker for predicting sperm freezability using spermatozoa collected from caudal epididymis. First, we measured the sperm parameters on epididymal spermatozoa collected from individual bulls. Subsequently, spermatozoa were categorized into two groups based on sperm motility; high freezing-tolerant spermatozoa (HFS) and low freezing-tolerant spermatozoa (LFS) groups. Second, we examined several sperm function parameters, such as motility, motion kinematics, viability, capacitation status, and mitochondrial membrane potential (MMP) between HFS and LFS groups. Finally, a comprehensive proteomic study was conducted to evaluate more specific biomarkers, capable of predicting HFS. Consequently, the difference of sperm protein concentration between HFS and LFS were searched in Pathway Studio program to foresee protein-protein interactions, protein related cellular function, and diseases association.

## Results

### Difference of sperm parameters between HFS and LFS

**Figure 1:**
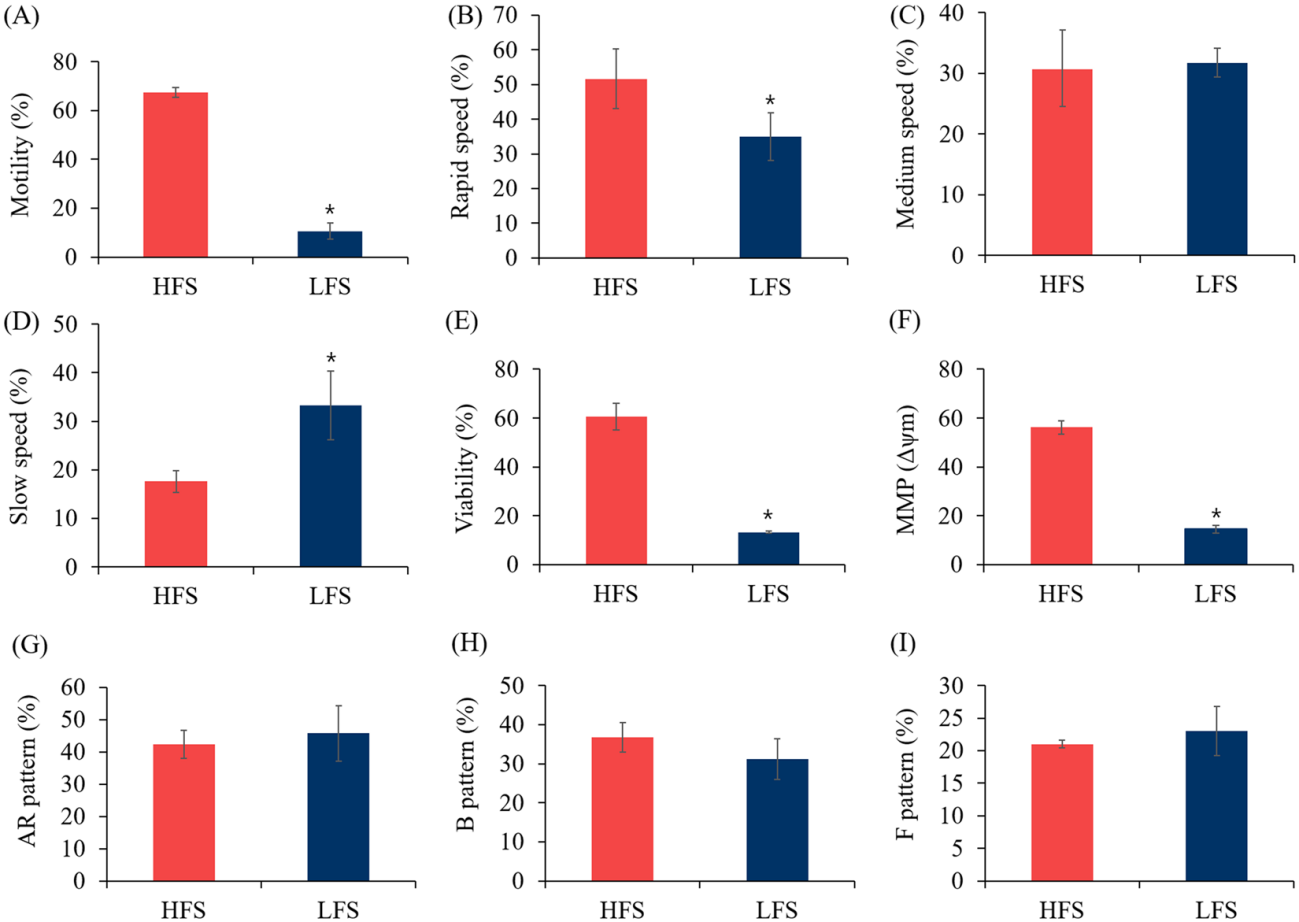
Differences of several sperm parameters. Percent differences in the levels of (**A**) motility, (**B**) rapid speed (>50 μm/s), (**C**) medium speed (25–50 μm/s), (**D**) slow speed (<25 μm/s) (**E**) viability, (**F**) MMP, (**G**) AR pattern, (**H**) F pattern, and (**I**) B pattern between HFS and LFS. Rapid, medium, and slow speed are expressed as a percentage of total motility. (AR pattern) acrosome-reacted, (B pattern) capacitated, and (F pattern) non-capacitated spermatozoa. The data expressed are the means of three experimental replicates with three sample per replicate. Data are presented as mean ± SEM. (*P < 0.05, calculated using two-tailed Student’s *t*-test).

CASA was conducted to investigate the differences of sperm motility, motion kinematics, and swimming speed in nine individual bull spermatozoa samples. Results showed that the three most highly motile spermatozoa had more than 60% motility, while the three least motile spermatozoa had less than 15% motility (*P* < 0.05, Fig. 1A). Based on this result, we categorized the spermatozoa into two groups (HFS and LFS). Various kinematic parameters, i.e. hyperactivated motility (HYP), curvilinear velocity (VCL), straight-line velocity (VSL), average path velocity (VAP), amplitude of head lateral displacement (ALH), linearity (LIN), and wobble (WOB) showed no significant differences between HFS and LFS (*P* > 0.05, Table 1). Additionally, rapid speed was significantly higher in HFS whereas slow speed was significantly higher in LFS. However, there is no difference of slow speed between HFS and LFS.

**Table 1:** Differences in the motion kinematics between HFS and LFS.

Parameters	HFS	LFS
VCL (μm/s)	53.05 ± 7.99	51.99 ± 4.27
VSL (μm/s)	33.64 ± 7.02	27.02 ± 4.89
VAP (μm/s)	40.60 ± 8.22	35.39 ± 4.10
ALH (μm)	2.44 ± 0.08	2.41 ± 0.38
LIN (%)	62.48 ± 3.84	50.21 ± 5.02
WOB (%)	75.48 ± 3.71	70.65 ± 0.83

Data presented as mean ± SEM. MOT = motility (%), HYP = hyperactivated motility; VCL = curvilinear velocity (µm/s), VSL = straight-line velocity (µm/s), VAP = average path velocity (µm/s), ALH = mean amplitude of head lateral displacement (µm), LIN (%) = linearity, and WOB (%) = wobble. The data expressed are the means of three experimental replicates with three sample per replicate. Data are presented as mean ± SEM.

Figure 1E,F shows the differences of sperm viability and MMP in both HFS and LFS. Our Results showed that number of viable spermatozoa was higher in HFS compared to LFS under osmotic condition (*P* < 0.05, Fig. 1E). In addition, to analyze the sperm MMP, which plays a central role by series of essential events, we performed rhodamine 123 staining. MMP was found to be significantly lower in LFS compared to HFS (*P* < 0.05, Fig. 1F).

To evaluate the differences of capacitation status between HFS and LFS, H33258***/***CTC dual staining method was performed. Our results showed that the percentage of live capacitated, non-capacitated, and acrosome-reacted spermatozoa have no differences between HFS and LFS (*P* > 0.05, Fig. 1G,H,I).

### Differences of sperm protein concentration between HFS and LFS

**Figure 2:**
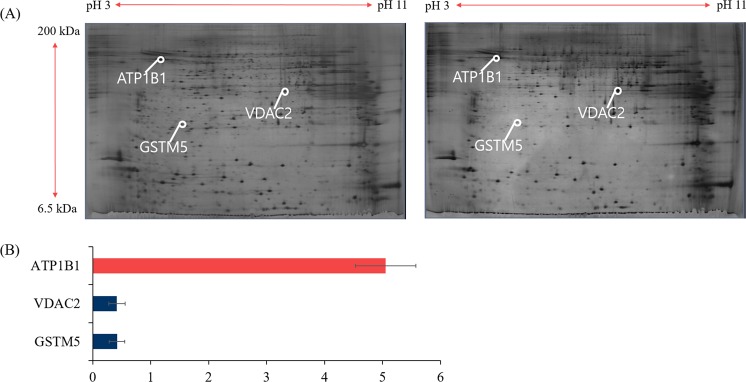
Representative 2DE gel image of protein separation. (**A**) Protein spots from HFS and LFS. (**B**) Relative quantification of VDAC2, GSTM5, and ATP1B1 in HFS vs. LFS observed by 2DE. VDAC2, and GSTM5 were higher in LFS. ATP1B1 was higher in HFS. Proteins with different concentration determined by at least two-fold changes between HFS and LFS. The data expressed are the means of three experimental replicates with three sample per replicate. Data are presented as mean ± SEM. (*P < 0.05, calculated using two-tailed Student’s *t*-test).

2DE was conducted to determine the different concentration of proteins in HFS and LFS following to cryopreservation. A total of 190 protein spots were identified, and 62 protein spots have different concentration among them (>2-fold). However, only three proteins were identified by ESI-MS/MS and MASCOT search. GSTM5 and VDAC2 showed high concentration in LFS, on the other hand ATP1B1 showed low concentration in HFS (*P* < 0.05, Fig. 2B, Table 2). The quantitative data of 62 protein spots are shown in the Supplementary Table 1.

**Table 2:** Proteins with different concentrations (>2-fold) between HFS and LFS.

Spot no.	NCBI no.	Protein	Symbol	MASCOT score^a^	Ratio of protein concentration^b^
3301	gi 23065563	Glutathione S-transferase Mu 5 (Fragment)	gstm5	59	0.42 ± 0.13
7501	gi 48146045	Voltage-dependent anion-selective channel protein 2	vdac2	327	0.42 ± 0.14
9937	gi| 28461221	ATP synthase subunit beta, mitochondrial	atp1b1	36	5.05 ± 0.52

^a^The MASCOT score is −10*Log(P), where P is the probability that the observed match is a random event. Individual ions score >33 indicates identity or extensive homology (*P* < 0.05). ^b^Ratio of protein concentration is the ratio of relative concentration of protein spots with LFS to HFS.

### Validation of proteins with different concentration between HFS and LFS

**Figure 3:**
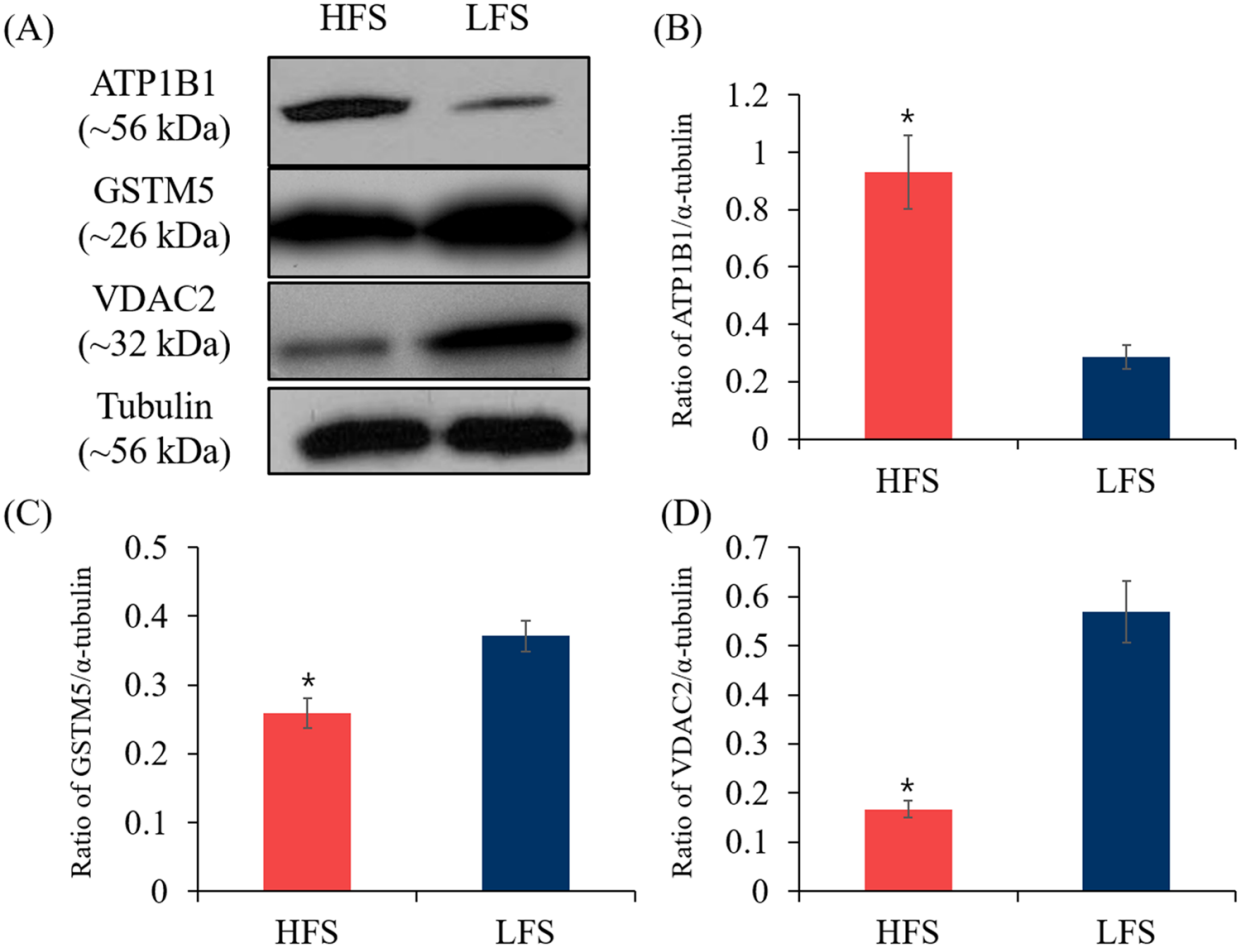
Concentration of VDAC2, GSTM5, and ATP1B1 in HFS and LFS. (**A**) Representative western blotting images of each protein in HFS and LFS. (**B**) The ratio of GSTM5 and α-tubulin in HFS and LFS. (**C**) The ratio of ATP1B1 and α-tubulin in HFS and LFS. (**D**) The ratio of VDAC2 and α-tubulin in HFS and LFS. The data expressed are the means of three experimental replicates with three sample per replicate. Data are presented as mean ± SEM. (*P < 0.05, calculated using two-tailed Student’s *t*-test). Representative uncropped images are shown in the Supplemental Figs 1–4.

To validate the 2DE results, western blotting analysis was conducted using commercial antibodies. GSTM5, VDAC2, and ATP1B1 were detected at ~26, ~32, and ~56 kDa, respectively. While GSTM5 and VDAC2 showed significantly high concentration in LFS, ATP1B1 showed significantly high concentration in HFS consistent with our results (*P* < 0.05, Fig. 3).

### Bioinformatics analysis of proteins with different concentration between HFS and LFS

**Figure 4:**
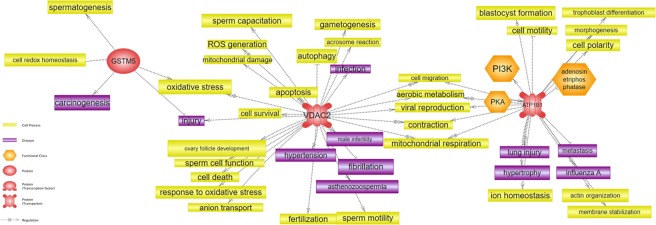
Signaling pathways of proteins with different concentration. Protein-protein interactions, cellular regulation, and associated diseases related to proteins were illustrated by Pathway Studio Program.

To investigate the cellular regulation, interacting proteins, and associated diseases between proteins, Pathway Studio program was used as a bioinformatics tool. We found that GSTM5 and VDAC2, which were higher in LFS, were involved with spermatogenesis, the acrosome reaction, and male infertility. ATP1B1, which are higher in HFS, was commonly associated with cell motility and blastocyst formation (Fig. 4).

## Discussion

Spermatozoa are not that resistant to the stresses caused by several factors, such as cold shock, osmotic stress, and ice crystal formation during cryopreservation^23,24^. These stresses have a detrimental effect on sperm structure and function, resulting in disruption of sperm physiology, which is directly associated with male fertility^11,16^. However, each spermatozoon has a different ability to protect against these stresses upon cryopreservation. Although many studies have been conducted to discover freezability markers, further studies with novel approaches are needed to overcome the limitations of cryopreservation.

Spermatozoa built in testes are not completely mature. Less mature spermatozoa must undergo several physiological and biological modifications to acquire motility or fertility from the caput to the caudal epididymis. Even though spermatozoa have partial maturity through the epididymis, the epididymis continues to suppress sperm motility up to ejaculation. Finally, spermatozoa are ejaculated with several mixtures, such as fluids of the prostate, seminal vesicles, and other accessory glands through the vas deferens^21,22^. During ejaculation, the composition of sperm proteins is modified by seminal plasma proteins, and eventually acquires resistance against many kinds of stresses, such as osmotic, heat, physical, oxidative, and freezing^16,21,22,25^. Therefore, it is worth studying the freezing biomarker using epididymal spermatozoa to discover the mechanism and proteomic effects on sperm freezability before ejaculation. Simultaneously, cryopreservation of epididymal spermatozoa might have a critical role to store the genetic information from the several cases, such as catastrophic injury and unexpected death^9,26^. In addition, several studies have shown that epididymal spermatozoa can replace the ejaculated spermatozoa for artificial insemination efficiently in equine industry^27,28^. Therefore, we used the epididymal spermatozoa for cryopreservation to demonstrate the specific role of freezability on sperm physiology, as well as to identify general markers related with freezing-tolerance.

Sperm motility and viability are widely considered to be the most important characteristic for the capacity of fertility and predicting freezability^11,29^. Therefore, we evaluated motility/motion kinematics, and viability in individual bull spermatozoa with different freezing abilities. Spermatozoa is less resistance to cryopreservation due to its high fluidity of the membrane, which can negatively affect the sperm structure and function^8,30^. Several studies demonstrated that sperm function, such as, motility and viability are affected by thermal shock with formation of intra/extracellular ice crystals during the freezing and thawing^31^. Along with the previous studies, our results showed that HFS are significantly more motile, fast and viable than LFS. Consistent with our findings, Rego *et al*.^16^ demonstrated that a high freezability group can more easily maintain their motility and viability than a low freezability group in bull ejaculated spermatozoa. Therefore, each bull has different sperm freezing tolerance, which affects sperm motility and viability.

It has been reported that lower ATP caused by disruption of mitochondrial activity is the critical reason for the decrease of sperm motility^29,32,33^. Furthermore, there is ample experimental evidence showing that low MMP has a positive correlation with not only loss of sperm motility, but also sperm viability^34–38^. Therefore, we measured MMP between HFS and LFS to better interpret the loss of sperm motility and viability. The intracellular MMP in HFS is significantly higher than in LFS. Based on our results, the damages that occur during the freezing/thawing could influence sperm motility and viability, perhaps as a consequence of levels of MMP. Subsequently, we evaluated the sperm capacitation status between HFS and LFS, which is a prerequisite for successful fertilization. Even though several studies demonstrated that freezing/thawing has an impact on capacitation status, we did not find significant differences on the sperm capacitation status between HFS and LFS^11,14^.

Many proteins are considered as biomarkers for the prediction of highly freezing tolerant spermatozoa obtained from ejaculated semen^15,39,40^. As we mentioned above, the composition of proteins in epididymal spermatozoa are completely different from ejaculated spermatozoa even when these were washed by Percoll. Therefore, 2DE using epididymal spermatozoa was conducted to discover more specific sperm protein biomarkers between HFS and LFS. Three proteins were identified between HFS and LFS. Subsequently, Pathway Studio was used to demonstrate the interactions and functions within these three proteins. Our results showed that VDAC2 and GSTM5 were higher in LFS rather than HFS, while ATP1B1 was significantly abundant in HFS.

Our proteomic study showed that the VDAC2 was different between HFS and LFS. VDAC2 is present in the mitochondrial, acrosomal, and dense fiber of spermatozoa^40,41^. This protein has a critical role in the transportation of ions and small molecules in spermatozoa through the lipid bilayer^42,43^. Several studies demonstrated that abnormal activity and alteration of this protein concentration have inimical effects on sperm function and fertility via alteration of ionic influx^44–46^. Since freezing/thawing step is associated with increased osmotic stress mediated via ionic imbalance, thus VDAC2 can be a candidate marker for predicting sperm freezability. Consistent with the current findings, VDAC2 also has reported as a freezability biomarker of ejaculated boar spermatozoa by another study^42^.

The stress response proteins GSTM5 also showed altered levels between LFS and HFS. GSTM5 is a member of the glutathione S-transferases (GST) mu class proteins, which play a critical role for protecting sperm function, such as motility, viability, and fertility, against oxidative stress^47,48^. As such, sperm resistance to oxidative stress at the freezing/thawing step of cryopreservation has a functional linkup with GSTM5 and thus could consider as a marker.

Another protein is ATP1B1 that was significantly higher in HFS then that of LFS. ATP1B1 is the subunit of ATP synthase that must undergo a conformational change in order for ATP synthase to obtain energy^49^. It has been reported that in the absence of ATP1B1, ATP synthase cannot synthesize ATP and the respiratory rate is decreased^50,51^. Further, inactivation of ATP synthase affects various sperm functions, including the MMP, intracellular ATP, and consequently motility/motion kinematics in stallion spermatozoa^52^. According to this evidence, high sperm motility and MMP in HFS might be the result of high concentration of ATP1B1 detected in our study. Therefore, increases ATP1B1 in HFS could maintain sperm motility and viability during freezing/thawing step on cryopreservation. Based on the current findings, we suggest that GSTM5, VDAC2, and ATP1B1 might be efficient biomarkers for predicting freezability. However, further studies are needed to examine the exact role of GSTM5, VDAC2, and ATP1B1 during cryopreservation.

In this study, we investigated the differences of sperm parameters between HFS and LFS. Simultaneously, we identified three candidate protein biomarkers that can be used to select spermatozoa with high resistance to cryopreservation. The current study is particularly important to store valuable genetic materials for the animal in the case of unexpected death/accident. However, further studies are needed to elucidate the underlying mechanism of the candidate biomarkers on sperm freezability.

## Materials and Methods

### Animal and spermatozoa collection

All procedures for sample preparation are in line with a method variation that was used by Yoon *et al*.^11^. Native Korean bull (beef cattle) testes were collected from a local slaughterhouse and transferred to laboratory on ice. The age range of the bulls at the time of slaughter were 30–36 months. In laboratory, spermatozoa were flushed out with phosphate-buffered saline (PBS, pH 7.4; Sigma-Aldrich, St. Louis, MO, USA) from the cut caudal epididymis of nine individual bulls. Flushed spermatozoa were washed at 700 × *g* for 15 min. All procedures were approved by the Institutional Animal Care and Use Committee (IACUC) of Chung-Ang University, Seoul, Republic of Korea. Experiments were performed according to the IACUC guidelines for the ethical treatment of animals.

### Cryopreservation of spermatozoa

Sperm cryopreservation was based on the protocol proposed by Awad and Graham^53^. Shortly, flushed spermatozoa were diluted to 100 × 10^6^ cells/mL in Tris-egg yolk buffer (TYB; 250 mM Tris, 88.5 mM citric acid, 68.8 mM glucose, and 20% egg yolk) and cooled to 4 °C over 2 h. Equal volumes of TYB with 12% glycerol was mixed to dilute the sample, which was then equilibrated at 4 °C for 2 h. Equilibrated samples were packaged into 0.5 ml straws and frozen in liquid nitrogen vapor for 15 min. Straws were stored into liquid nitrogen for 2 weeks. After 2 weeks, samples were thawed at 37 °C for 1 min.

### Computer-assisted sperm analysis (CASA) for individual bull spermatozoa

Thawed sperm motility and kinematic parameters were measured by using the CASA system (SAIS Plus version 10.1; Medical supply, Seoul, Korea)^54^. Briefly, 10 μL of the spermatozoa were placed in a 37 °C Makler chamber (Makler, Haifa, Israel). Spermatozoa were observed using a 10× phase contrast objective, and analyzed by SAIS software. The program setting was established (frames acquired, 20; frame rate, 30 Hz; minimum contrast, 7; minimum size, 5; low/high size gates, 0.4–1.5; low/high intensity gates, 0.4–1.5; non-motile head size, 16; non-motile brightness, 14). Hyper-activated (HYP) spermatozoa were measured as curvilinear velocity (VCL) ≥150 µm/sec, mean amplitude of head lateral displacement (ALH) ≥5 µm/sec, and linearity (LIN) ≤50% as reported previously^29,55,56^. Sperm speed parameters were classified into rapid (>50 μm/s), medium (25–50 μm/s), and slow speed (<25 μm/s). Individual bull spermatozoa were categorized into HFS (≥60%) and LFS (≤15%) groups based on sperm motility. All sperm speed parameters are expressed as percentage of total motility. For each of the three independent replicate experiments, three samples were used.

### Hypo-osmotic swelling test (HOST)

To evaluate sperm viability and membrane integrity between HFS and LFS, a hypo-osmotic swelling test (HOST) was performed. Briefly, samples were washed with PBS and adjusted to a concentration of 5 × 10^6^ cells/mL. Spermatozoa were mixed with HOST solution (distilled water: 0.9% NaCl, 150 mOsm/kg), and then incubated at 37 °C for 30 min. After incubation, samples were smeared on microscope slides and fixed with a fresh fixative (methanol:acetic acid [3:1, v/v]). Spermatozoa were observed using a Microphot-FXA microscope (Nikon, Osaka, Japan) with a 20× objective (Nikon, Osaka, Japan). Sperm swelling patterns were classified broadly as viable or nonviable according to the 2010 WHO guidelines. For each of the three independent replicate experiments, three samples were used.

### Evaluation of mitochondrial membrane potential (MMP)

MMP in HFS and LFS was measured using rhodamine 123 (Rh123; Sigma-Aldrich, St. Louis, MO, USA). Briefly, spermatozoa were mixed with 1 μM Rh123 diluted in PBS and the concentration of spermatozoa were adjusted to 5 × 10^6^ cells/mL, and then incubated at 37 °C for 15 min. Fluorescence intensity of Rh123 was measured by flow cytometry (Dual-Laser FACS Aria II, BD Biosciences, San Jose, CA, USA) with 488 nm excitation and 525 nm emission wavelengths and analyzed. For each of the three independent replicate experiments, three samples were used.

### Combined H33258/chlortetracycline fluorescence (H33258/CTC) assessment of spermatozoa

H33258***/***CTC dual staining assays were conducted to examine the capacitation status of spermatozoa between HFS and LFS using a dual-staining method^46,57^. Shortly, 15 μL of H33258 solution were added to 135 μL of samples, and incubated for 10 min at room temperature. 250 μL of 2% polyvinylpyrrolidone in Dulbecco’s phosphate-buffered saline (DPBS) was added, and the mixture was centrifuged at 100 × *g* for 2.5 min. The supernatant was removed and 100 μL of DPBS and CTC solution were added into pellet. Capacitation status was detected using a Microphot-FXA microscope with ultraviolet BP 340–380/LP 425 and BP 450–490/LP 515 excitation/emission filters for H33258 and CTC, respectively. Four different type of patterns were observed; dead (D pattern, blue fluorescence), non-capacitated (F pattern, bright yellow fluorescence presented evenly over the entire sperm head), capacitated (B pattern, bright yellow fluorescence presented over the acrosomal region and a dark post-acrosomal region), or acrosome-reacted (AR pattern, no fluorescence over the head, or yellow fluorescence only in the post-acrosomal region) as previously reported^29,58^. For each of the three independent replicate experiments, three samples were used.

### Two-dimensional gel electrophoresis (2DE) and image analysis

To examine proteins with different concentrations, thawed spermatozoa from HFS and LFS were analyzed by two-dimensional gel electrophoresis (2DE). All procedures for sample preparation followed the method of Kwon *et al*.^59^. Samples were washed at 700 × *g* for 15 min on isotonic 45% Percoll in PBS. The motile spermatozoa were subsequently added into rehydration buffer [7 M urea, 2 M thiourea, 4% (w/v) CHAPS (USB, Cleveland, OH, USA), 0.05% (v/v) Triton X-100, 1% (w/v) octyl β-D-glucopyranoside, 24 μM PMSF, 1% (w/v) DTT, 0.5% (v/v) IPG buffer, and 0.002% (w/v) bromophenol blue] at 4 °C for 1 h. The solubilized proteins were rehydrated with 24-cm-long NL Immobiline DryStrips (Amersham Biosciences, Piscataway, NJ, USA) at 4 °C for 12 h, and developed by first dimension electrophoresis by using an IPGphor isoelectric focusing device. After equilibration, second-dimension electrophoresis was conducted with 12.5% (w/v) sodium dodecyl sulfate-polyacrylamide gel electrophoresis (SDS-PAGE) gels. Silver staining was performed to stain the gels according to manufacturer’s instructions (Amersham Biosciences). Each gel was scanned with a GS-800 calibrated scanner (Bio-Rad, Hercules, CA, USA) to analyze the matched spots between HFS and LFS. Detected spots were analyzed using the PD Quest 8.0 software (Bio-Rad). For each of the three independent replicate experiments, three samples were used.

### Nano ESI-MS/MS of sperm proteins

Analysis of the peptides, which were acquired by in-gel trypsin digestion, was conducted by nano-electrospray ionization (ESI) using a MicroQ-TOF2 III mass spectrometer (AB Sciex Instruments). The MS/MS data were determined in the ion search option in the Mascot 2.4 software and SwissProt FASTA. Homology search DB was used for finding the peptide fragment files. Individual ions score > 33 indicate identity or extensive homology (*P* < 0.05).

### Western blot analysis of ESI-MS/MS data

Western blot analysis of ESI-MS/MS data in HFS and LFS were performed as previously described^46,57^. Briefly, thawed spermatozoa from HFS and LFS were adjusted to a concentration of 1 × 10^8^ cells/mL. Each group of spermatozoa were washed with DPBS and centrifuged at 10,000 × *g* for 10 min, and resuspended in Laemmli sample buffer (63 mM Tris, 10% glycerol, 10% SDS, and 5% bromophenol blue) containing 5% β-mercaptoethanol for 5 min. After incubation, degraded proteins from HFS and LFS were separated with a 12% SDS-PAGE gel for electrophoresis and were transferred to a polyvinylidene fluoride (PVDF) membrane (Amersham Biosciences). The membrane was blocked with 5% blocking solution (3%; Amersham Biosciences) for 1 h at room temperature. After blocking, the membrane was incubated overnight at 4 °C with primary antibodies diluted in blocking solution (1:1,000). The primary antibodies used were rabbit polyclonal anti-Glutathione S-Transferase Mu 5 (GSTM5), anti-ATPase synthase subunit beta (ATP1B1), anti-voltage-dependent anion-selective channel protein 2 (VDAC2), and mouse monoclonal anti-α tubulin antibodies. The membrane was washed five times in PBS-T, and was subsequently incubated with the horseradish peroxidase (HRP)-conjugated goat anti-rabbit IgG or mouse IgG secondary antibody for 1 h at room temperature. After washing, protein-antibody complexes were detected by enhanced chemiluminescence reagent. All protein bands were scanned using a GS-800 calibrated imaging densitometer (Bio-Rad) and quantified by Quantity One program (v. 4.6, Bio-Rad). The ratios of GSTM5/α-tubulin, ATP1B1/α-tubulin and VDAC2/α-tubulin were calculated. All antibodies were purchased from Abcam (Cambridge, MA, USA) except the above mentioned. For each of the three independent replicate experiments, three samples were used.

### Bioinformatics analysis of proteins with different concentration

Pathway Studio program (Elsevier, Amsterdam, The Netherlands) was used to discover protein-protein interactions, cellular regulation, and disease associations for proteins between HFS and LFS group. After inserting GSTM5, ATP1B1, and VDAC2 as input objects, we searched such parameters related with different sperm protein concentration.

### Statistical analysis

Data were analyzed using Student’s two-tailed *t*-test with SPSS statistical software (version 12.0; Chicago, IL, USA). Differences between the control and treated samples were considered significant at *p* values less than 0.05. Data are presented as mean ± SEM.

## Supplementary information

Dataset 1
