# Supplementary material for: Freezability biomarkers in bull epididymal spermatozoa
Source: Sci Rep. 2019 Sep 5;9:12797. doi: 10.1038/s41598-019-49378-5 (PMC6728342; doi:10.1038/s41598-019-49378-5)
Supplement: Supplementary file 1 — Dataset 1 [file 41598_2019_49378_MOESM1_ESM.docx]

SUPPLEMENTAL MATERIAL

**Freezability biomarkers in bull epididymal spermatozoa**

Do-Yeal Ryu^1,2^, Won-Hee Song^1,2^_,_ Won-Ki Pang^1,2^, Sung-Jae Yoon^1,2^,

Md Saidur Rahman^1,2^ and Myung-Geol Pang^1,2*^

^1^Department of Animal Science and Technology, Chung-Ang University, Anseong, Gyeonggi-do 456-756, Republic of Korea

^2^BET Research Institute, Chung-Ang University, Anseong, Gyeonggi-do 17546, Republic of Korea

*Correspondence should be addressed to: Myung-Geol Pang (mgpang@cau.ac.kr)

Tel: +82.31.670.4841; Fax: +82.31.675.9001


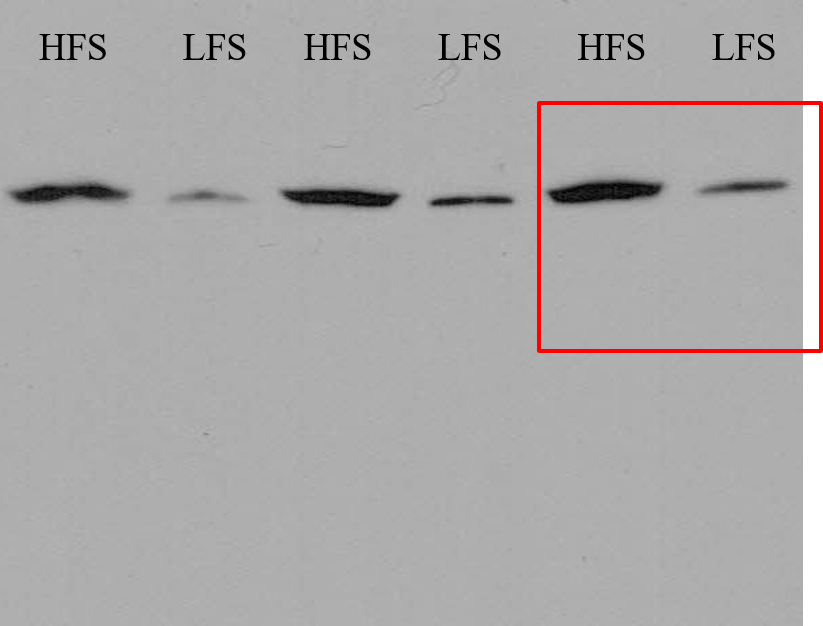


**Figure S1.** Uncropped image of western blot represents ATP1B1


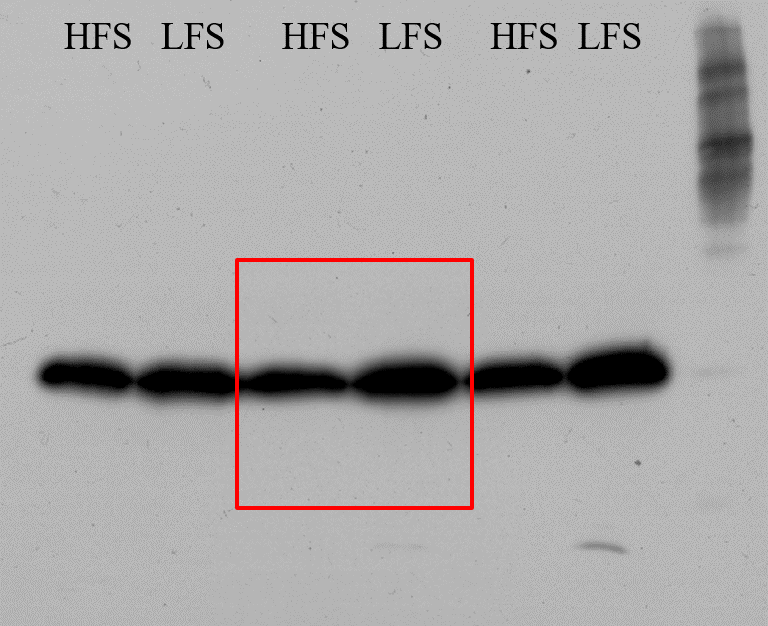


**Figure S2.** Uncropped image of western blot represents GSTM5


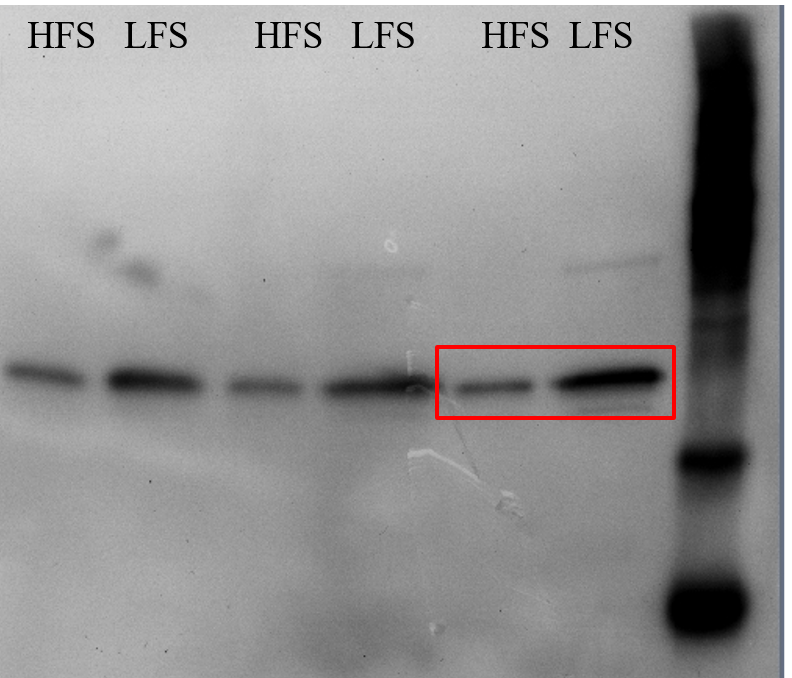


**Figure S3.** Uncropped image of western blot represents VDAC2


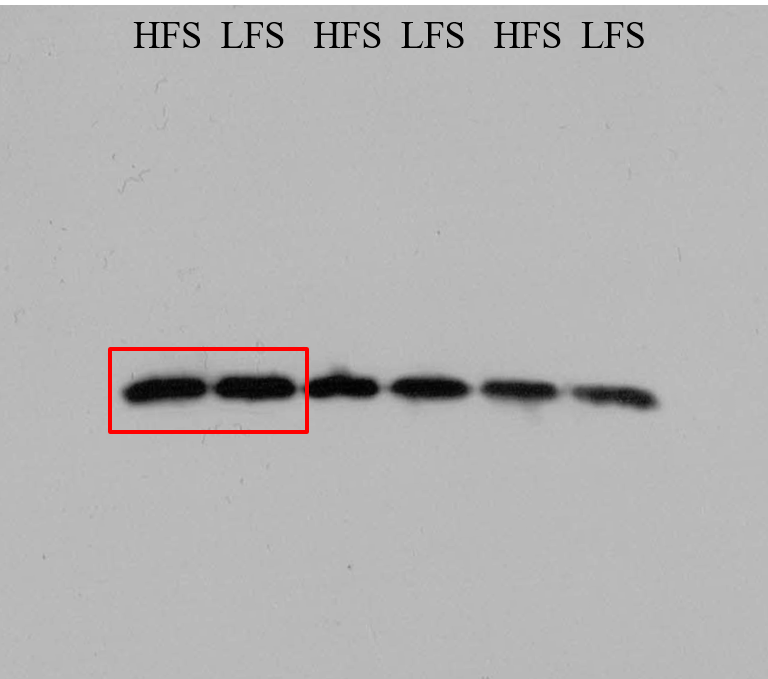


**Figure S4.** Uncropped image of western blot represents tubulin

**Figure S5.** Uncropped image of 2DE represents high freezing-tolerant spermatozoa group (1^st^ replication).


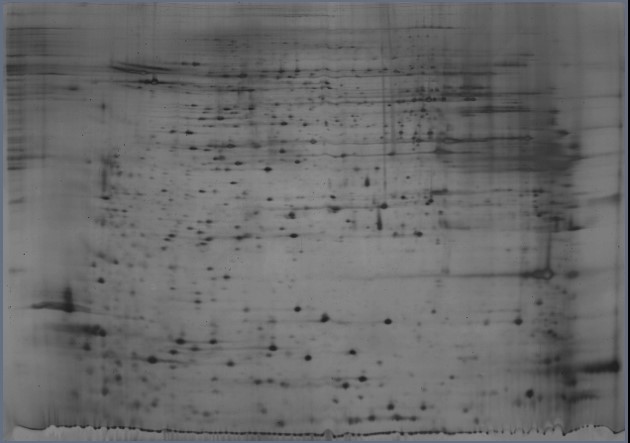


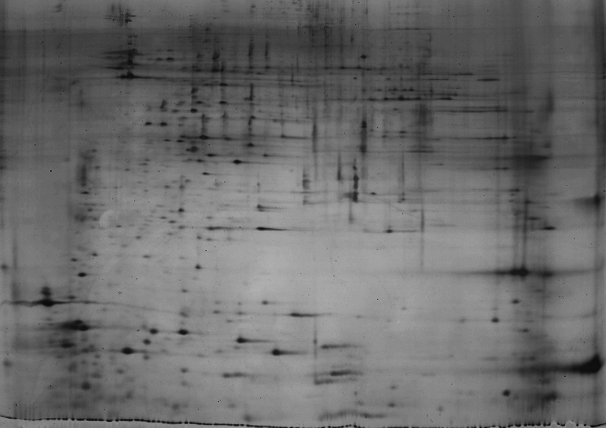


**Figure S6.** Uncropped image of 2DE represents high freezing-tolerant spermatozoa group (2^nd^ replication).


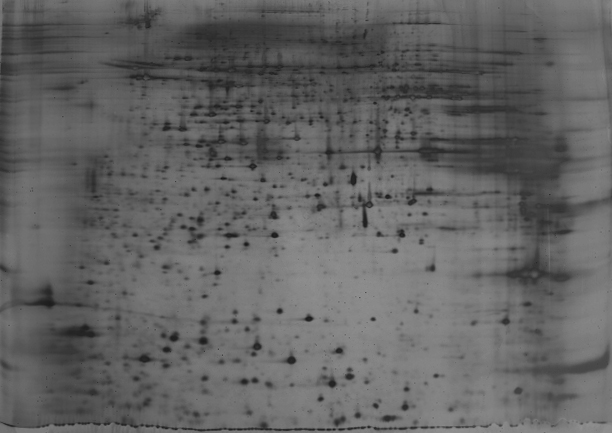


**Figure S7.** Uncropped image of 2DE represents high freezing-tolerant spermatozoa group (3^rd^ replication).


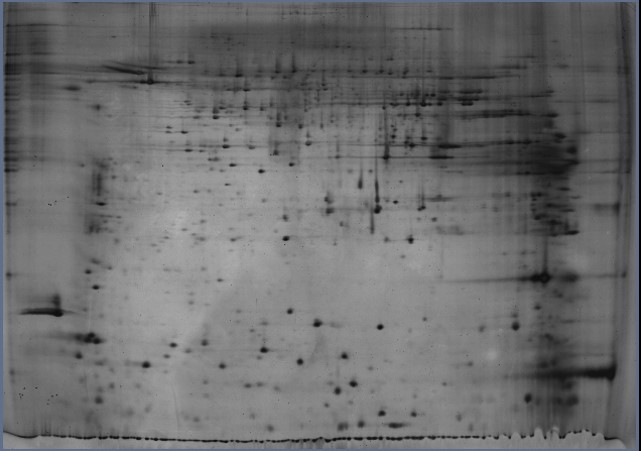


**Figure S8.** Uncropped image of 2DE represents low freezing-tolerant spermatozoa group (1^st^ replication).


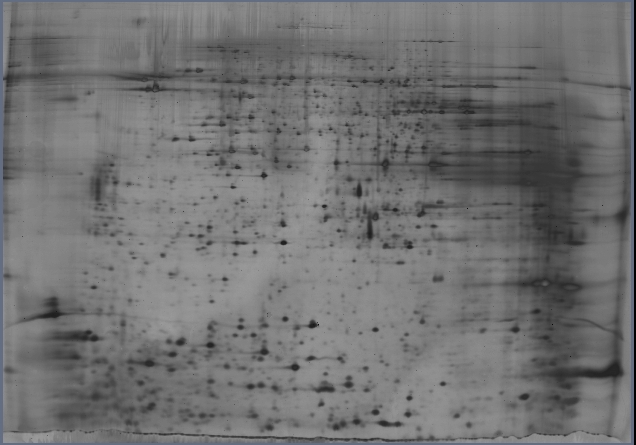


**Figure S9.** Uncropped image of 2DE represents low freezing-tolerant spermatozoa group (2^nd^ replication).


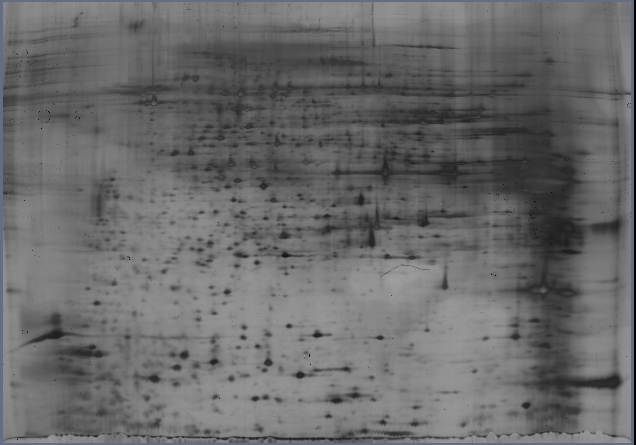


**Figure S10.** Uncropped image of 2DE represents low freezing-tolerant spermatozoa group (3^rd^ replication).

**Supplementary table 1.** Protein spots (62) with different concentrations (>2-fold) between HFS and LFS

| Spot number | HFS1/LFS1 | HFS2/LFS2 | HFS3/LFS3 | AVERAGE | STDEV | SEM |
| --- | --- | --- | --- | --- | --- | --- |
| 9305 | 1.705084746 | 183.8333333 | 44 | 76.51280603 | 95.31781916 | 55.031769 |
| 1201 | 10.96761134 | 12.37710438 | 3.287197232 | 8.877304315 | 4.892202015 | 2.8245142 |
| 5401 | 7.469298246 | 3.276595745 | 5.224489796 | 5.323461262 | 2.098102733 | 1.2113402 |
| 9937 | 5.818181818 | 5.277955272 | 4.055299539 | 5.050478876 | 0.903187475 | 0.5214555 |
| 9101 | 11.26862745 | 2.73902237 | 1.114696486 | 5.040782102 | 5.454278313 | 3.1490291 |
| 8706 | 3.94017094 | 4.198653199 | 2.84030837 | 3.659710836 | 0.721296451 | 0.4164407 |
| 8602 | 2.558541267 | 2.652892562 | 5.084269663 | 3.431901164 | 1.431770507 | 0.8266331 |
| 402 | 3.138263666 | 6.807228916 | 0.331278891 | 3.425590491 | 3.247522088 | 1.8749578 |
| 5901 | 5.760309278 | 1.62041116 | 1.72005772 | 3.033592719 | 2.361931361 | 1.3636617 |
| 7602 | 2.00330033 | 2.68297456 | 3.971061093 | 2.885778661 | 0.999433706 | 0.5770233 |
| 5402 | 2.763565891 | 2.26645768 | 3.112745098 | 2.714256223 | 0.425293053 | 0.2455431 |
| 8405 | 3.059065934 | 1.043024772 | 3.330882353 | 2.477657686 | 1.249839886 | 0.7215954 |
| 7603 | 1.965827338 | 3.985849057 | 1.246520875 | 2.39939909 | 1.420200011 | 0.8199529 |
| 1417 | 2.298245614 | 2.4 | 2.158878505 | 2.28570804 | 0.121048697 | 0.0698875 |
| 7704 | 1.329192547 | 2.076086957 | 3.09047619 | 2.165251898 | 0.884020819 | 0.5103897 |
| 6901 | 2.130434783 | 2.219298246 | 1.988038278 | 2.112590435 | 0.116658085 | 0.0673526 |
| 2305 | 2.086309524 | 2.199275362 | 1.926345609 | 2.070643498 | 0.137137635 | 0.0791765 |
| 1406 | 2.186868687 | 1.950892857 | 1.954869359 | 2.030876968 | 0.135107422 | 0.0780043 |
| 7301 | 1.493066256 | 3.058921162 | 1.493913043 | 2.015300154 | 0.903802404 | 0.5218106 |
| 2104 | 0.946141479 | 0.155532359 | 0.385488959 | 0.495720932 | 0.406668183 | 0.23479 |
| 2408 | 0.367396594 | 0.046511628 | 1.046875 | 0.486927741 | 0.510781255 | 0.2948997 |
| 2405 | 0.09403255 | 0.047058824 | 1.316901408 | 0.485997594 | 0.719967009 | 0.4156731 |
| 3504 | 0.285560345 | 0.14686623 | 1.019946809 | 0.484124461 | 0.46918888 | 0.2708863 |
| 4102 | 0.624231782 | 0.235213831 | 0.585702681 | 0.481716098 | 0.214344696 | 0.123752 |
| 3401 | 0.521276596 | 0.541401274 | 0.37037037 | 0.477682747 | 0.093478395 | 0.0539698 |
| 3402 | 0.319148936 | 0.177993528 | 0.924324324 | 0.473822263 | 0.396478616 | 0.228907 |
| 1403 | 0.4875 | 0.39084507 | 0.518248175 | 0.465531082 | 0.066482049 | 0.0383834 |
| 7706 | 0.319389764 | 0.391891892 | 0.678074866 | 0.463118841 | 0.189654184 | 0.1094969 |
| 2302 | 0.485163205 | 0.469006721 | 0.427263479 | 0.460477802 | 0.029877272 | 0.0172497 |
| 1305 | 0.483146067 | 0.760964912 | 0.130612245 | 0.458241075 | 0.315913462 | 0.1823927 |
| 2306 | 0.636826783 | 0.124463519 | 0.601515972 | 0.454268758 | 0.286164874 | 0.1652174 |
| 4104 | 0.354444444 | 0.622004357 | 0.377799416 | 0.451416073 | 0.148194588 | 0.0855602 |
| 7107 | 0.85915493 | 0.075085324 | 0.407035176 | 0.44709181 | 0.393566623 | 0.2272258 |
| 4301 | 0.099532398 | 0.324357405 | 0.905178211 | 0.443022671 | 0.415725123 | 0.240019 |
| 1502 | 0.42043222 | 0.397260274 | 0.457705678 | 0.425132724 | 0.030495619 | 0.0176067 |
| 1407 | 0.673267327 | 0.128540305 | 0.460471567 | 0.420759733 | 0.274526236 | 0.1584978 |
| 3301 | 0.420950533 | 0.190283401 | 0.647193585 | 0.41947584 | 0.228458662 | 0.1319007 |
| 7501 | 0.359518349 | 0.208058608 | 0.687061823 | 0.418212926 | 0.244836292 | 0.1413563 |
| 2403 | 0.202020202 | 0.180916976 | 0.831357049 | 0.404764742 | 0.369590427 | 0.2133831 |
| 2106 | 0.450271248 | 0.080229226 | 0.672932331 | 0.401144268 | 0.299389942 | 0.1728529 |
| 3304 | 0.361134279 | 0.219809322 | 0.618073316 | 0.399672306 | 0.201909477 | 0.1165725 |
| 5501 | 0.216205983 | 0.183908046 | 0.767631774 | 0.389248601 | 0.32808712 | 0.1894212 |
| 3307 | 0.168 | 0.45 | 0.538461538 | 0.385487179 | 0.193473166 | 0.1117018 |
| 1301 | 0.130067568 | 0.42800789 | 0.587378641 | 0.381818033 | 0.232128158 | 0.1340193 |
| 3303 | 0.398496241 | 0.253164557 | 0.493793103 | 0.381817967 | 0.121178166 | 0.0699622 |
| 3403 | 0.12170088 | 0.274647887 | 0.734729494 | 0.377026087 | 0.319079915 | 0.1842209 |
| 2105 | 0.290052356 | 0.314871795 | 0.52523172 | 0.376718624 | 0.129213412 | 0.0746014 |
| 7401 | 0.071358749 | 0.881533101 | 0.1625 | 0.371797283 | 0.44379008 | 0.2562223 |
| 2604 | 0.263297872 | 0.5 | 0.342857143 | 0.368718338 | 0.120451548 | 0.0695427 |
| 1409 | 0.368035191 | 0.125408942 | 0.558252427 | 0.35056552 | 0.216949908 | 0.1252561 |
| 6102 | 0.159257175 | 0.473760933 | 0.414832926 | 0.349283678 | 0.167184573 | 0.0965241 |
| 1309 | 0.227864583 | 0.179516686 | 0.583981337 | 0.330454202 | 0.220887723 | 0.1275296 |
| 3302 | 0.177298311 | 0.271345876 | 0.502659574 | 0.317101254 | 0.167437015 | 0.0966698 |
| 2502 | 0.3 | 0.175438596 | 0.468085106 | 0.314507901 | 0.146861684 | 0.0847906 |
| 3305 | 0.160526316 | 0.297343616 | 0.47768595 | 0.311851961 | 0.159076798 | 0.091843 |
| 2301 | 0.042372881 | 0.263702172 | 0.619489559 | 0.308521537 | 0.291157168 | 0.1680997 |
| 1103 | 0.056470588 | 0.012867647 | 0.811342593 | 0.293560276 | 0.448942312 | 0.259197 |
| 4402 | 0.177705977 | 0.189208129 | 0.513736264 | 0.293550123 | 0.190773497 | 0.1101431 |
| 3201 | 0.041322314 | 0.470588235 | 0.345918367 | 0.285942972 | 0.2208282 | 0.1274952 |
| 6701 | 0.405555556 | 0.137529138 | 0.2997543 | 0.280946331 | 0.134999426 | 0.077942 |
| 1302 | 0.164772727 | 0.127413127 | 0.5 | 0.264061952 | 0.205180425 | 0.118461 |
| 1411 | 0.19047619 | 0.1 | 0.324675325 | 0.205050505 | 0.113044497 | 0.0652663 |

HFS = High freezing-tolerant spermatozoa, LFS = Low freezing-tolerant spermatozoa, STDEV = Standard deviation , and SEM = Standard error of mean.
